# Thyroid cancer complicating familial adenomatous polyposis: mutation spectrum of at-risk individuals

**DOI:** 10.1186/1897-4287-11-13

**Published:** 2013-10-05

**Authors:** Seth Septer, Voytek Slowik, Ryan Morgan, Hongying Dai, Thomas Attard

**Affiliations:** 1 Section of Pediatric Gastroenterology, Children’s Mercy Hospital, Kansas City, MO, USA; 2 Section of Pediatrics, Children’s Mercy Hospital, Kansas City, MO, USA; 3 Department of Medical Research, Children’s Mercy Hospital, Kansas City, MO, USA; 4 Georgetown University, Georgetown, Washington DC, USA

**Keywords:** Thyroid cancer, Familial adenomatous polyposis, FAP, Adenomatous polyposis gene mutation, APC, Papillary thyroid carcinoma

## Abstract

**Background:**

Lifetime risk of thyroid cancer associated with FAP has been reported as 1-2%. The mean age at diagnosis of thyroid carcinoma in FAP has been reported at 28 years. The aims of this paper are to better understand gene mutations associated with thyroid cancer and refine surveillance recommendations for patients with FAP.

**Methods:**

We performed a search in Pubmed, Ovid Medline and Embase with the terms ("Thyroid Gland"[Mesh] OR "Thyroid Neoplasms"[Mesh]) AND "Adenomatous Polyposis Coli"[Meshdenomatous Polyposis Coli"[Mesh] to identify subjects with thyroid cancer and FAP. As a reference group for APC mutations in the unselected FAP population, we used the UMD-APC database referenced in the Orphanet portal, which includes APC mutation data on 2040 individuals with FAP.

**Results:**

There were 115 reported cases of thyroid cancer in patients with FAP (95 female: 11 male) with an average age of 29.2 years. Gene mutation testing results were reported in 48 patients. On comparing the prevalence of APC mutation in the population of FAP patients with thyroid cancer and the prevalence of the same mutation in the reference population an increased odds ratio was evident in individuals harboring an APC mutation at codon 1061 (OR: CI 4.1: 1.7-8.9). Analysis of the prevalence of thyroid cancer in individuals with FAP segregated by the region of the gene affected shows an increased risk of thyroid cancer in individuals harboring mutations proximal to codon 512 (OR 2.6, p 0.0099).

**Conclusions:**

There is increased risk for thyroid cancer in individuals with APC mutations at the 5' end (proximal to codon 528) along with the established high risk group harboring mutation at codon 1061. It is suggested that these patients might benefit from directed surveillance by annual ultrasound from age 18 years onwards.

## Introduction

Familial Adenomatous Polyposis (FAP) is an autosomal dominant syndrome with a predisposition for colorectal cancer. FAP is characterized by profuse adenomatous polyposis in the colon and rectum with nearly 100% lifetime risk of colorectal cancer. Approximately 1% of all cases of colorectal cancer each year in the United States are due to FAP [1]. Without prophylactic colectomy, the mean age of colorectal cancer is 39 years and the mean life expectancy is 42 years [2]. Colorectal involvement in the classic form of FAP typically presents in childhood or early adolescence whereas a milder pattern of colorectal involvement (Attenuated FAP) may present later in life with a later age at presentation of colon cancer.

**Figure 1:**
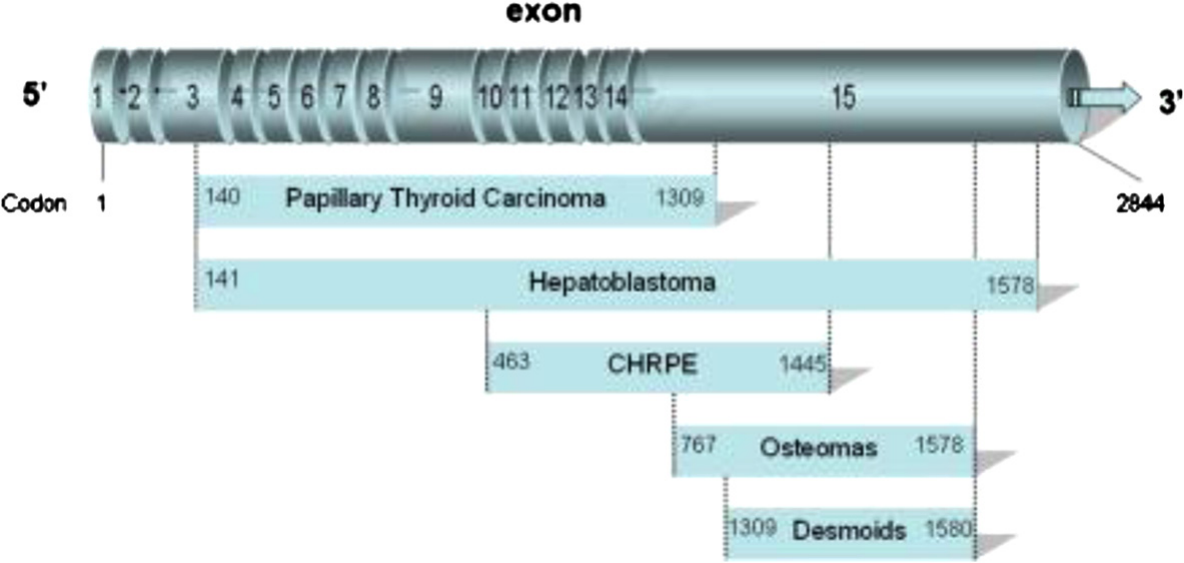
**Diagram of APC gene showing genotype-phenotype correlations of extra-intestinal manifestations of FAP as depicted by Groen et al. **[21]**.** With kind permission from Springer Science and Business Media.

Most patients with FAP harbor a germline mutation in the adenomatous polyposis coli (*APC*) gene on chromosome 5q21. The majority of *APC* mutations are either frameshift or nonsense mutations resulting in a truncated protein [3]. Penetrance is nearly complete for the colonic phenotype but is variable for extra-colonic manifestations of the disease. The *APC* gene encodes a tumor suppressor protein consisting of 2843 amino acids. More than 60% of *APC* mutations are found in the mutation cluster region (MCR) between codons 1284 and 1580 [4], or 1284–1464 [5]. The two most frequently described germline mutations are located at codon 1309 (c3927_3931delAAAGA) and codon 1061 (c.3183_87delACAAA) [6] Disease expression in FAP is to some extent dependent on the specific *APC* mutations (genotype-phenotype correlation; Figure 1).

Extra-intestinal manifestations are common in FAP; children are also at risk and thus need to be assessed regularly from birth. Extra-colonic manifestations in children with FAP may include a 750–7500 fold increased risk of hepatoblastoma [7,8] as well as an increased risk of medulloblastoma [9], osteomas, supernumerary teeth or missing teeth, congenital hypertrophy of retinal pigment epithelium (CHRPE), desmoid tumors and fibromas. Several referral centers have adopted an extra-intestinal tumor surveillance strategy that includes reported genotype-phenotype correlations [10,11].

Thyroid cancer in a patient with FAP was first described by Crail in 1949 [12]. In this group, lifetime risk of thyroid cancer has been reported as 1-2% [13-17]. This may be even higher in certain kindreds as Herraiz et al. reported thyroid carcinoma in 12% of 51 patients with FAP [18]. Thyroid cancer observed in FAP is typically the cribiform-morular variant of papillary thyroid carcinoma (PTC) [19]. The mean age at diagnosis of thyroid carcinoma in FAP has been reported at 25–28 years [16,20] with one third concomitant diagnosis, one third first diagnosis of FAP and one third diagnosed first with thyroid cancer [16,20].

While the female:male ratio in sporadic cases of PTC is 3–4:1, the magnitude of the disparity appears to be even higher in PTC associated with FAP. In such patients, the female predominance is reported to be 10–17:1 [6,21]. Accordingly, female patients with FAP have been estimated to have a 100 to 160 fold risk of thyroid cancer compared to normal individuals [14,22] with peak incidence less than 30 years of age. Although an increased risk is recognized, clinical surveillance guidelines for PTC in FAP patient’s remains poorly defined. Most centers advise annual clinical examination with consideration for ultrasound. Guidelines from a previous paper [21] suggest looking for palpable nodules at least yearly and referral to an endocrinologist for fine needle aspiration (FNA) if nodules are present [23]. Several authors have recommended liberal use of thyroid ultrasound in FAP patients [18,24]. Others have recommended FNA for thyroid nodules greater than 1 cm in size [25,26].

Phenotype-genotype correlation of the development of thyroid cancer in FAP has been attempted before but the observations remain conflicting. Previous literature has stated that papillary thyroid carcinoma is more prevalent in codons 140–1309 (Figure 1). Thirteen of fifteen patients with thyroid carcinoma and FAP had mutations in the *APC* gene between codons 778 and 1309 in a study by Cetta [27]. Another study [15] screened 16 patients with both FAP and PTC. Germline *APC* gene mutations were noted in 12 of 13 patients tested; in nine of them the mutation was located before codon 1286, and outside the mutation cluster region (MCR). Harb and coworkers [28] also reiterated the majority of *APC* mutations associated with PTC appear to be proximal to the MCR, although there appears to be significant overlap with the *APC* gene region (codons 463–1387) associated with congenital hypertrophy of the retinal pigment epithelium (CHRPE) [21,29,30].

This study is a systematic review of the genotype-phenotype associations of thyroid cancer in patients with FAP. Literature search was undertaken to find all cases of thyroid malignancy that had a described mutation in the *APC* gene. This was further refined by controlling for the frequency distribution of mutations in a reference database of *APC* gene mutations in FAP patients. The aims are to better understand gene mutations associated with thyroid cancer and to better use specific *APC* gene mutations to refine surveillance recommendations.

## Methods

We performed a search in Pubmed, Ovid Medline and Embase with the terms ("Thyroid Gland"[Mesh] OR "Thyroid Neoplasms"[Mesh]) AND "Adenomatous Polyposis Coli"[Mesh] NOT "Comment"[Publication Type] OR "Letter"[Publication Type] OR "Editorial"[publication type]). The reference lists of the retrieved articles were also reviewed to identify additional studies, as were review articles on the subject.

The peer reviewed articles identified through the initial search were reviewed and the authors independently abstracted information from each of these studies. Each study was reviewed for sample size, diagnosis of FAP, diagnosis of TC and documentation of *APC* mutation. Studies with incomplete or duplicate patient reports were excluded. The remaining patients with information relating to gender, age at onset of thyroid cancer or during illness and specific mutations of *APC* gene were accrued to a database for further evaluation.

As a reference group for *APC* mutations in the unselected FAP population, we used the UMD-*APC* database referenced in the Orphanet portal (http://www.umd.be/APC/). This includes data on 2040 individuals with FAP including their *APC* gene mutation.

Fisher’s exact test was performed to compare the mutation rate between reference and thyroid cancer patients with mutation. Odds ratio and 95% exact confidence interval were determined. The 95% confidence interval of significant odds ratio does not cross 1. Statistical significance was claimed with p < 0.05. All statistical analyses were performed in SAS 9.2 (Cary NC).

## Results

The initial search resulted in 49 articles related to thyroid cancer and FAP. After review, 18 studies were determined as meeting inclusion criteria. These eighteen studies included a total of one hundred fifteen reported cases of thyroid cancer in patients with FAP. Affected individuals were mainly female (96 F: 11 M) with an average age of 29.2 (+/− 10.3) years.

*APC* gene mutation testing was reported in 48 patients (Table 1). The reference population of individuals with FAP included 2040 individuals. We compared the prevalence of *APC* mutations in the published patients with thyroid cancer to the prevalence of the same mutation in the reference population (see Table 1). An increased odds ratio for thyroid cancer was evident for mutations at codon 1061 (OR: CI 4.1: 1.7-8.9). Mutations at codon 1309 conferred less risk (0.8: 0.2-2.0). Without adjusting for the reference population prevalence, individuals with mutations at codon 1061 (18.7%) and 1309 (10.42%) were most frequently identified with thyroid cancer. However, these two mutations were also the most common *APC* mutations in the reference population accounting for 5.3% and 12.9% of cases respectively. Using this comparison mutations in codon 1309 were actually less common as a percentage of the total in the FAP-TC group than in the FAP reference group.

**Table 1:** Comparison of APC mutation frequency in reference population of individuals with FAP, with APC mutation frequency in reported cases of thyroid cancer in individuals with FAP – including comparison of regional mutation frequency

**Codon**	**Mutation frequency in RP**	**TC cases**	**TC frequency of mutation**	**Odds ratio (CI) by region**	**OR (CI)**	**p-value**
140	0.05%	1	2.08%	**2.6** (1.2-5.1) **p = 0.0099**	43.4 (0.5-3410.0)	0.0454
157	0.15%	1	2.08%		14.5 (0.3-183)	NS
159	0.05%	1	2.08%		43.4 (0.5-3410.0)	0.0454
175	0.05%	1	2.08%		43.4 (0.5-3410.0)	0.0454
180	0%	1	2.08%		∞ (2.2-∞)	0.0230
278	0.39%	2	4.17%		11.1(1.1-57.3)	0.0207
302	0.64%	1	2.08%		3.3 (0.1-23.0)	NS
312	0%	2	4.17%		∞ (12.4-∞)	0.0005
499	0.34%	1	2.08%		6.2 (0.1-49.6)	NS
512	0%	1	2.08%		∞ (2.2-∞)	0.0230
528	0.05%	2	4.17%	1.6 (0.7-3.4) p = 0.1906	88.7 (4.5-5237.0)	0.0015
564	0.69%	2	4.17%		6.3 (0.7-28.6)	NS
593	0.25%	1	2.08%		8.7 (0.2 - 79.4)	NS
698	0.05%	3	6.25%		136.1 (10.5-7145.1)	0.00004
778	0.15%	1	2.08%		14.5 (0.3-183)	NS
938	0.10%	1	2.08%	**0.4** (0.2-0.8) **p = 0.0033**	21.7 (0.4-420.8)	NS
976	0.05%	1	2.08%		43.4 (0.5-3410.0)	0.0454
993	0.10%	1	2.08%		21.7 (0.4-420.8)	NS
**1061**	**5.34%**	**9**	**18.75%**		**4.1 (1.7-8.9)**	**0.0011**
1068	0.83%	2	4.17%		5.2 (0.6-22.8)	NS
1105	0.10%	1	2.08%		21.7 (0.4-420.8)	NS
1110	0.25%	1	2.08%		8.7 (0.2 - 79.4)	NS
1275	0%	1	2.08%		∞ (2.2-∞)	0.0230
**1309**	**12.89%**	**5**	**10.42%**		**0.8 (0.2-2.0)**	**NS**
1464	0.59%	1	2.08%		3.6 (0.1-25.2)	NS
1948	0%	2	4.17%		∞ (12.4-∞)	0.0005
2092	0%	2	4.17%		∞ (12.4-∞)	0.0005
		48				

RP = reference population (from UMD/APC database).

TC = thyroid cancer.

NS = not significant.

CI = confidence interval.

Bold= statistically significant odds ratio by region, or bold to highlight the most common APC mutations.

Further analysis of the prevalence of thyroid cancer in individuals with FAP segregated by the region of the gene affected shows an increased risk of thyroid cancer in individuals harboring mutations proximal to codon 512 (OR 2.6, p 0.0099). An intermediate risk was found for individuals harboring mutation in codons 513 through 937 (OR 1.6, p NS) and the lowest risk is in individuals with mutation distal to codon 938 (OR 0.4, p 0.0033) even when including all cases with the high risk codon 1061 (Table 1).

## Discussion

To our knowledge this is the largest group of pooled cases of thyroid cancer in patients with FAP including *APC* mutation data reported to date. This study corroborates earlier observations that thyroid cancers in patients with FAP are more prevalent in younger females. The mean age at onset (29.2 years) was similar to that previously reported. This is, however, the first analysis that attempts to define the actual risk of thyroid cancer associated with specific *APC* mutations in FAP patients whilst adjusting for the frequency distribution of *APC* mutations in FAP patients.

Our observations expand on work previously reported by Cetta et al. [20] which compared the difference in incidence of germline mutations before and after codon 1220 between 317 FAP patients without PTC and 24 patients with FAP and PTC. They reported a significant difference (p = 0.005), with FAP and PTC more common in those with mutations proximal to codon 1220. The authors then suggested that mutations at 5’ end of exon 15 may direct PTC screening in patients with FAP. Using the same arbitrary cutoff, the same difference was found in our group, which comprised a larger reference and FAP-PTC group (p = 0.0001).

This study shows that there is an increased risk of thyroid cancer associated with *APC* mutation in codon 1061; this is true both in absolute numbers of patients reported as well as when adjusted to the frequency of *APC* mutations at this locus in an unselected population. This observation is in line with prior observations although our analysis suggests that the degree of risk conferred is modest in comparison with other, less common mutations. In fact, although 24% of reported cases of thyroid cancer in FAP harbored a mutation proximal to codon 513, this range of mutations accounts for only 11.45% of the population with FAP from the UMD-*APC* database. Conversely, 82.4% of FAP patients from this database were reported with mutation at the 3’ end; distal to codon 938, but only 56.2% of the reported cases of thyroid cancer localized to the same region, paradoxically suggesting that individuals harboring mutations within that spectrum are relatively low risk although in absolute terms of occurrence they are reported more often. This observation also raises consideration for the need to re-examiner some other recommendations on phenotype-genotype correlations that are of even greater clinical relevance. These include the mutation spectrums associated with desmoids, hepatoblastoma, medulloblastoma and upper gastrointestinal involvement in individuals with FAP.

Genotype-directed disease surveillance has repeatedly been proposed as a potential strategy geared toward the detection of early intestinal and extra-colonic malignancy in FAP. The increased relative risk for TC in young, female patients with FAP has been previously noted and is corroborated in our data. Whether the specific *APC* mutation prompts different PTC surveillance techniques and schedules in certain groups of patients has not been previously determined.

Previous authors have recommended surveillance including yearly thyroid exams [14,31] or ultrasound [18,32] for all patients with FAP. As noted by Herraiz et al. [18], palpable thyroid nodules are appreciated in 5% of women and 1% of men in the general population [33,34], while the prevalence of thyroid nodules detected by high-resolution ultrasound is 19-67% in randomly selected individuals [35]. Thus, it appears a portion of nodules will not be appreciated by physical exam only. Ultrasound examination is clearly superior to physical examination alone [36], and if we can extrapolate from epidemiologic observations in the general population earlier detection of lesions will translate in better patient outcomes [37]. Although proof of improved outcomes from targeted, more intensive surveillance in this specific population is lacking, our observations support annual ultrasound examination in female patients, 18 years of age and older with FAP harboring mutation at codon 1061, or any mutation proximal to codon 528, followed by fine needle aspiration when indicated. Continued annual palpation of the neck as is established standard of care for clinical surveillance for thyroid masses should be continued in all other patients, with a low threshold for subsequent ultrasound. The more intensive surveillance for thyroid involvement in this population seems justified in view of the younger age at diagnosis and more aggressive behavior reported in syndromic compared with sporadic thyroid cancer including higher rates of reoperation and death in the syndromic cases [38].

This study has several limitations; it is a pooled metanalysis of a group of publications ranging from registry based reports, case series and case reports. Thyroid malignancy in FAP was not the focus of most of the included studies. Although efforts were made to exclude duplicate studies through careful scrutiny of publications from the same author, group or center we cannot be sure that individual patients were not included in more than one registry or seen at more than one center resulting in over-representation in the final dataset. This appears to have been unlikely given that duplicate – identical clinical data could not be found in the final dataset. The study also necessarily suffers from the inclusion of studies over a period of time during which testing modalities for *APC* mutations has changed with test results (egs. segmental involvement of *APC* with codon involvement through sequencing) and results in attrition of the data that may not be comparable. It is noteworthy that our search revealed only 48 published patients with FAP, a specified *APC* gene mutation and thyroid cancer. This underscores the need for a common resource for the pooling of mutation analysis and clinical features of this and other relatively rare polyposis syndromes through expanded use of registries.

In conclusion, our study reiterates the importance of continued clinical vigilance for thyroid cancer in younger women with FAP. Building upon earlier studies however, we emphasize the increased risk in individuals with mutations at the 5’ end (proximal to codon 528) along with the established high risk group harboring mutation at codon 1061. It is suggested that these patients might benefit from directed surveillance by annual ultrasound from age 18 years onwards.

## Abbreviations

FAP: Familial adenomatous polyposis; APC: Adenomatous polyposis coli; MCR: Mutation cluster region; PTC: Papillary thyroid cancer; CHRPE: Congenital hypertrophy of the retinal pigment epithelium; FNA: Fine needle aspiration; TC: Thyroid cancer.

## Competing interests

The authors have no competing interests to declare.

## Authors’ contributions

Study Concept and Design- TA and SS. Data Acquisition- VS, RM, SS. Analysis and Interpretation- HD, SS, TA, VS. Drafting of Manuscript- SS, VS, TA. Revision and Approval of Manuscript- All authors.
